# In this issue of *Current Oncology*

**Published:** 2009-01

**Authors:** M. McLean

In this editorial for the first issue of *Current Oncology*’s sixteenth year, I am pleased to begin with the important announcement that Dr. Martin Chasen has graciously accepted the position of managing editor. His assumption of this role will allow the journal to process its substantially increased quantity of submissions in a more timely manner. Everyone is working hard to achieve a turnaround measurable in weeks rather than in months. Welcome, Dr. Chasen!

The major thrust of *Current Oncology* continues to be the field of education, and in this issue, readers will find manuscripts from Dr. Nahum Sonenberg and colleagues discussing the feasibility of targeted therapy for mtor-dependent tumours with specific inhibitors (perhaps timely, in view of our soon-to-be-published supplement on developments surrounding the management of renal cell cancer). In a similar vein, Dr. Paul Williams and colleagues use the Updates and Developments in Oncology section to discuss the concept of “genomacy,” the development and validation of potential genomic drug response predictor models, made increasing complex in the era of combination therapies.

Dr. Peter Ellis and colleagues report on their efforts to develop consensus recommendations on the use of epidermal growth factor receptor tyrosine kinase inhibitors in non-small-cell lung cancer—a challenging task given the sheer number of studies (often with confusing messages) that have been published of late. The importance of this review is reflected in the effects of the new global data on patient care and provincial budgets.

As we, the Editorial Board, reiterate our ongoing thanks to contributors who write topical reviews by request, we are also particularly encouraged by the increasing visibility of the journal and the growth in its perceived value as a venue for the publication of original clinical research. To ensure that *Current Oncology* continues to develop into a leading force in the cancer field is our collective goal.

